# Supplementary figures and images for: HPTMT Parallel Operators for High Performance Data Science and Data Engineering
Source: Front Big Data. 2022 Feb 7;4:756041. doi: 10.3389/fdata.2021.756041 (PMC8860100; doi:10.3389/fdata.2021.756041)

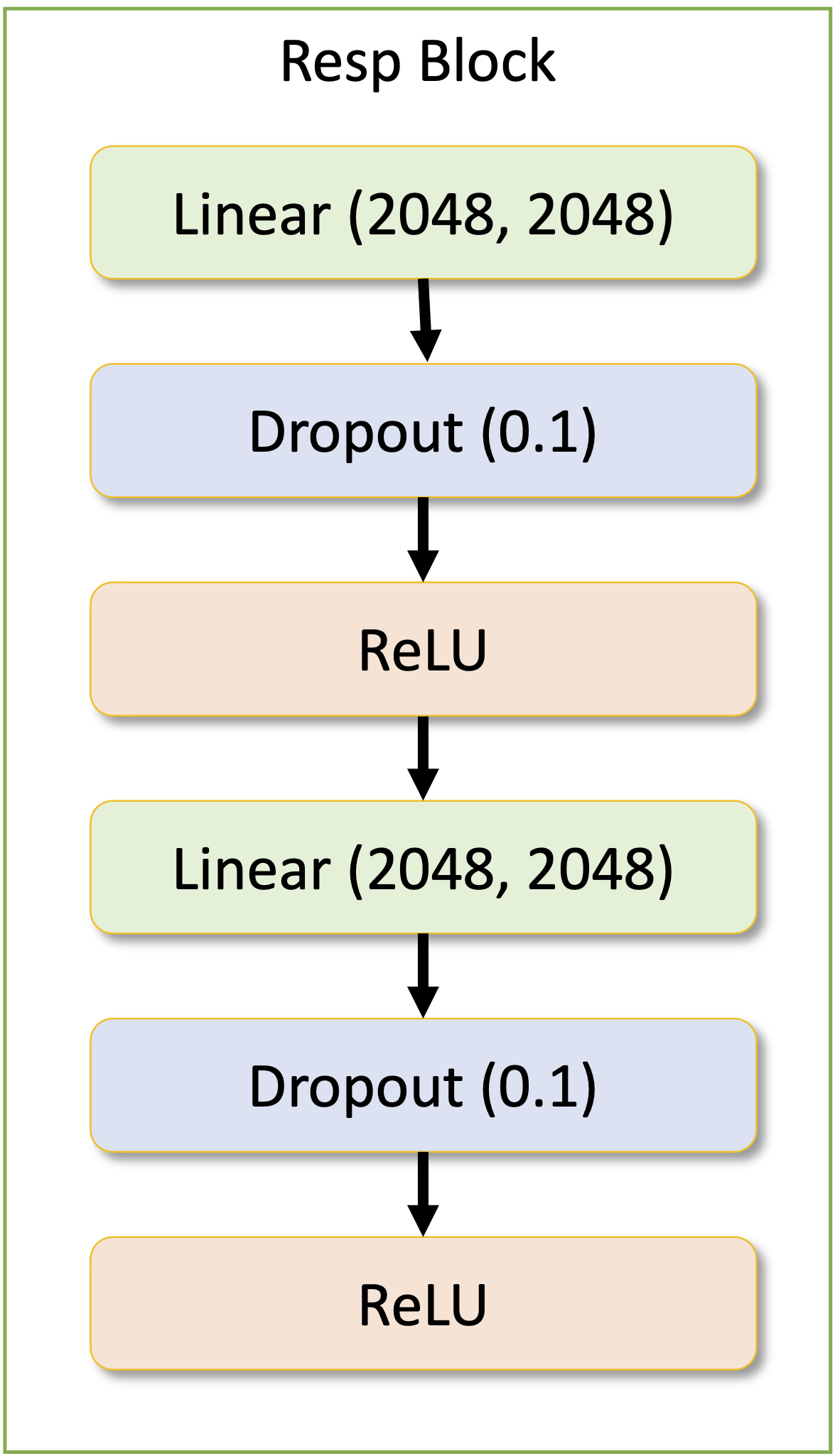

Supplement: Supplementary file 1 [file Image1.TIFF]

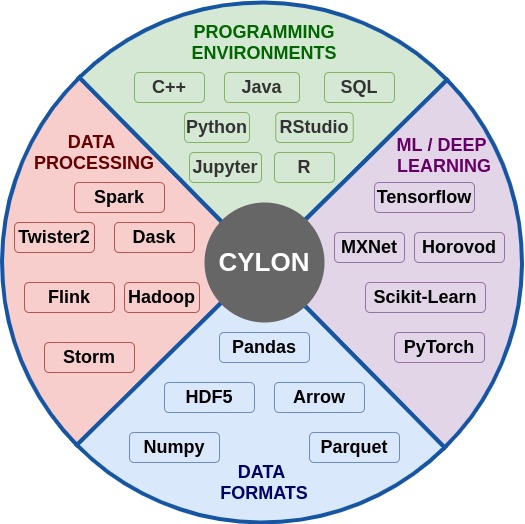

Supplement: Supplementary file 2 [file Image2.JPEG]
